# Detecting *Nothofagus pumilio* Growth Reductions Induced by Past Spring Frosts at the Northern Patagonian Andes

**DOI:** 10.3389/fpls.2019.01413

**Published:** 2019-10-31

**Authors:** Gabriel Sangüesa-Barreda, Ricardo Villalba, Vicente Rozas, Duncan A. Christie, José Miguel Olano

**Affiliations:** ^1^EiFAB-iuFOR, Universidad de Valladolid, Soria, Spain; ^2^Laboratorio de Dendrocronología e Historia Ambiental, Instituto Argentino de Nivología, Glaciología y Ciencias Ambientales (IANIGLA), CONICET, Mendoza, Argentina; ^3^Laboratorio de Dendrocronología y Cambio Global, Facultad de Ciencias Forestales y Recursos Naturales, Universidad Austral de Chile, Valdivia, Chile; ^4^Center of Climate and Resilience Research (CR)^2^, Santiago, Chile

**Keywords:** climate change, dendroecology, extreme event, frost damage, temperature pattern, tree rings, warm spring

## Abstract

Extreme climatic events, such as late frosts in spring during leaf flush, have considerable impacts on the radial growth of temperate broadleaf trees. Albeit, all broadleaved species are potentially vulnerable, damage depends on the particularities of the local climate, the species, and its phenology. The impact of late spring frosts has been widely investigated in the Northern Hemisphere, but the potential incidence in Southern Hemisphere tree species is still poorly known. Here, we reconstruct spring frost occurrence at 30 stands of the deciduous tree *Nothofagus pumilio* in its northern range of distribution in the Patagonian Andes. We identified tree ring-width reductions at stand level not associated with regional or local drought events, matching unusual minimum spring temperatures during leaf unfolding. Several spring frosts were identified along the northern distribution of *N. pumilio*, being more frequent in the more continental Argentinean forests. Spring frost in 1980 had the largest spatial extent. The spring frosts in 1980 and 1992 also induced damages in regional orchards. Spring frost damage was associated with (i) a period of unusually warm temperatures at the beginning of leaf unfolding, followed by (ii) freezing temperatures. This study helps expand our understanding of the climatic constraints that could determine the future growth and dynamics of Andean deciduous forests and the potential use of tree-rings as archives of extreme events of spring frosts in northern Patagonia.

## Introduction

Disturbances, such as fires, insect outbreaks, or droughts, dictate the future dynamics of forest ecosystems around the world, and their occurrence and severity will increase with climate change (Seidl et al., 2017; Navarro et al., 2018). The nature, timing, and impacts of natural disturbances are dissimilar, but their interaction in a warmer climate may result in nonlinear responses (Buma, 2015). Furthermore, punctual but acute disturbances in short time windows, but coincident with key periods for tree species performance, may trigger unpredictable impacts. In this sense, spring climatic conditions largely determine the annual carbon gains of many temperate species (Saurer et al., 2003). Extreme climatic events in spring such as frosts, heat waves or droughts, act as selective forces (Inouye, 2000; Pau et al., 2011; Vanoni et al., 2016) constraining key stages in tree dynamics (Hufkens et al., 2012).

In temperate deciduous species, spring-frost episodes at the beginning of leaf unfolding damage new tissues through the formation of intracellular ice crystals in buds and new leaves, deviating carbon reserves to replace the damaged tissues (Inouye, 2000) and resulting in a shorter growing season in response to the retreat of the leaf-out (Gu et al., 2008). These events cause lower annual secondary growth (Dittmar et al., 2006;Príncipe et al., 2017) and impact negatively on reproduction and population dynamics (Inouye, 2000; Augspurger, 2011). Determining the frequency and severity of these extreme climate events is essential to assess their influence on deciduous forest functioning and dynamics and predict their interaction with a warmer climate (Augspurger, 2013).

The timing of leaf unfolding represents an evolutionary trade-off between maximizing carbon acquisition in the growing season and escaping from frost damage (Lockhart, 1983; Leinonen and Hänninen, 2002; Gömöry and Paule, 2011). Vulnerability to spring frost damage depends primarily on species-specific safeguarding strategies (early to late-leafing species; Bigler and Bugmann, 2018) but also on intraspecific variability between- and within-population in leafing phenology (Baliuckas et al., 2005; Salmela et al., 2013). Leaf unfolding depends also on yearly climatic conditions, and there is a broad agreement that the timing of leaf unfolding has moved ahead in many temperate deciduous species in response to warmer spring temperatures (Menzel and Fabian, 1999; Parmesan and Yohe, 2003; Menzel et al., 2006; Jeong et al., 2011; Fu et al., 2015). An extended vegetative period may increase forest productivity (i.e., higher photosynthetic gains with larger biomass and secondary growth), but also reduce the safety margins to avoid frost events during late-spring, as has been observed in diverse mountain ecosystems (Bigler and Bugmann, 2018; Augspurger, 2009; Augspurger, 2013; Vitasse et al., 2018).

Since observed rate of warming tends to increase with elevation, high-elevation forests are more vulnerable to late frosts due to a relatively more important advance in the timing of leaf unfolding and narrower safety margins (Bigler and Bugmann, 2018; Vitasse et al., 2018). The balance between these processes, frost episodes and longer growing seasons due to warmer springs, may regulate the long-term dynamics of mixed forests (Hufkens et al., 2012), including the distribution range of deciduous species (Muffler et al., 2016). Current climatic projections simulate larger intra-annual temperature fluctuations in temperate latitudes (IPCC, 2013), and thus, the damaging frost effects could also occur later, prolonging the critical period. A scenario of warmer springs and larger temperature fluctuations may lead to greater risks of frost damage (the so-called “frost-damage hypothesis”; Morin and Chuine, 2014), but this question remains under debate and more observational data across species distribution are indeed needed to test this hypothesis (but see Augspurger, 2013).

Deciduous species showing large spatial distributions are potentially vulnerable to experiencing damage by freezing temperatures at some point in their life (Inouye 2000). In this context, several questions emerge: “How frequently do the frost damage events occur?” “When do they happen?” and “Where do they occur?” It is expected that the impacts will not be homogeneous throughout the species’ range due to peculiarities of the local climate (i.e. thermal amplitude) or of the forest (i.e. high *vs.* low elevation sites), and the phenological timing shifts associated with such factors (Charrier et al., 2015). Impacts in remote areas may be unnoticed since induced defoliations by frosts are only visible during a short period from frost damage until refoliation. Fortunately, the unusually lower radial growth is a permanent footprint which can help to reconstruct a freezing occurrence (Ningre and Colin, 2007; Dittmar et al., 2006; Príncipe et al., 2017). However, other high-frequency disturbances such as droughts or insect outbreaks could also induce similar pattern of growth (Paritsis and Veblen, 2011; Rodríguez-Catón et al., 2015; Rodríguez-Catón et al., 2016). Since reconstructing the occurrence of these episodes is key to identifying the response of forests to climate change and establish the historical range of frost disturbances, we need to develop methodologies to discern frost episodes retrospectively from other high-frequency growth reducing events.

The occurrence of late frost episodes is well-documented in the Northern Hemisphere, with studies in the eastern coast North America (e.g., Gu et al., 2008; Hufkens et al., 2012), and in Europe, particularly with *Fagus sylvatica* L. (e.g., Menzel et al., 2015; Dittmar et al., 2006; Príncipe et al., 2017), but also with a wider range of woody species (Bigler and Bugmann, 2018). However, to the best of our knowledge, no work has evaluated the incidence of late frost defoliation in temperate deciduous forests in the Southern Hemisphere. In this research, we explore these events on *Nothofagus pumilio* (Poepp et Endl.) Krasser, the dominant subalpine tree species in the Patagonian Andes. *N. pumilio* shows a wide latitudinal range of distribution across the southern Andes covering diverse bioclimatic gradients, from warmer and drier in the north to cooler and wetter conditions in the south, and from wetter to drier environments along the west-to-east precipitation gradient across the Andes (Villalba et al., 2003). The wide distribution and altitudinal range makes *N. pumilio* potentially sensitive to late frost damages (Villalba et al., 1997). Nevertheless, information on spring frost damages on *N. pumilio* is lacking, although in lowland agricultural areas in northern Patagonia frost damage is a major concern for economically important fruit trees (Pascale et al., 1997; Tassara 2007; Cittadini et al., 2006).

Here, we use a tree-ring network of 30 chronologies across the northern *N. pumilio* distribution area in Chile and Argentina to evaluate the incidence of spring frost damage. We use multiple, independent, and complementary methods to reconstruct the frequency of frost impacts in *N. pumilio* based on spring minimum temperature records (driver) and tree-ring information (proxy). Our main goals are: (i) to determine which years and stands have experienced growth reductions caused by spring-frost episodes; and (ii) to identify those areas more vulnerable to spring frost damage. Our initial hypothesis is that *N. pumilio* experiences late-spring frosts at its northern distribution area, as reported for temperate deciduous species in Northern Hemisphere (e.g., Gu et al., 2008; Bigler and Bugmann, 2018). We also hypothesize that the frequency of frost events increases toward the eastern slopes of the Andes due to their larger thermal amplitude in spring and the occurrence of more frequent frost events.

## Materials and Methods

### Study Species and Study Area

*N. pumilio* is a deciduous tree species encompassing a large latitudinal range between 35°S in Central Chile and 55°S in Tierra del Fuego. In northern Patagonia, it grows from c.a. 1000 m a.s.l. to c.a. 1700 m a.s.l. in the upper tree line (Veblen et al., 1996).

To compare with *N. pumilio,* we use *Austrocedrus chilesis* (D. Don) Pic. Serm. et Bizarri a coexisting evergreen conifer that is unaffected by spring frost damage in the leaves, *A. chilensis* is sensitive to extreme minimum temperatures in the xylem at juvenile stages (up to c.a. 120 years; Muñoz-Salazar et al., in prep.), when thin bark does not prevent frost damage (Payette et al., 2010; Arco Molina et al., 2016). However, frost rings are not related to lower radial growth (Payette et al., 2010). Although there are no specific studies about temperature threshold damaging leaves in *A. chilensis*, this species inhabits the thermal zone VIII (−6.7°C to −12.1°C; USDA-United States Department of Agriculture) where evergreen species tolerate temperatures between −13°C and −35°C (Bannister and Neuner, 2001).

We focused our study on the northern distribution area of *N. pumilio* between 35°S and 42°S in both eastern (Argentina) and western (Chile) slopes of the Andes in northern Patagonia. In this area, insect outbreaks (e.g., *Ormiscores* spp.) are less frequent than in southern populations of *N. pumilio* (Paritsis and Veblen, 2011). The windward side of the Andes intercepts wet westerly winds conforming a marked west-east gradient of decreasing precipitation from over 3000 mm yr^−1^ in Chile to below 800 mm yr^−1^ in the Argentinean leeward side (Barros et al., 1983). Daily thermal amplitude increases as distance from Pacific Ocean increases. The mean annual temperature ranges from 6°C at upper *N. pumilio* forests to 8°C at the foothills in Argentina and 11°C in Chile (Lara et al., 2001; Villalba et al., 2003).

### Tree-Ring Chronologies

**Figure 1:**
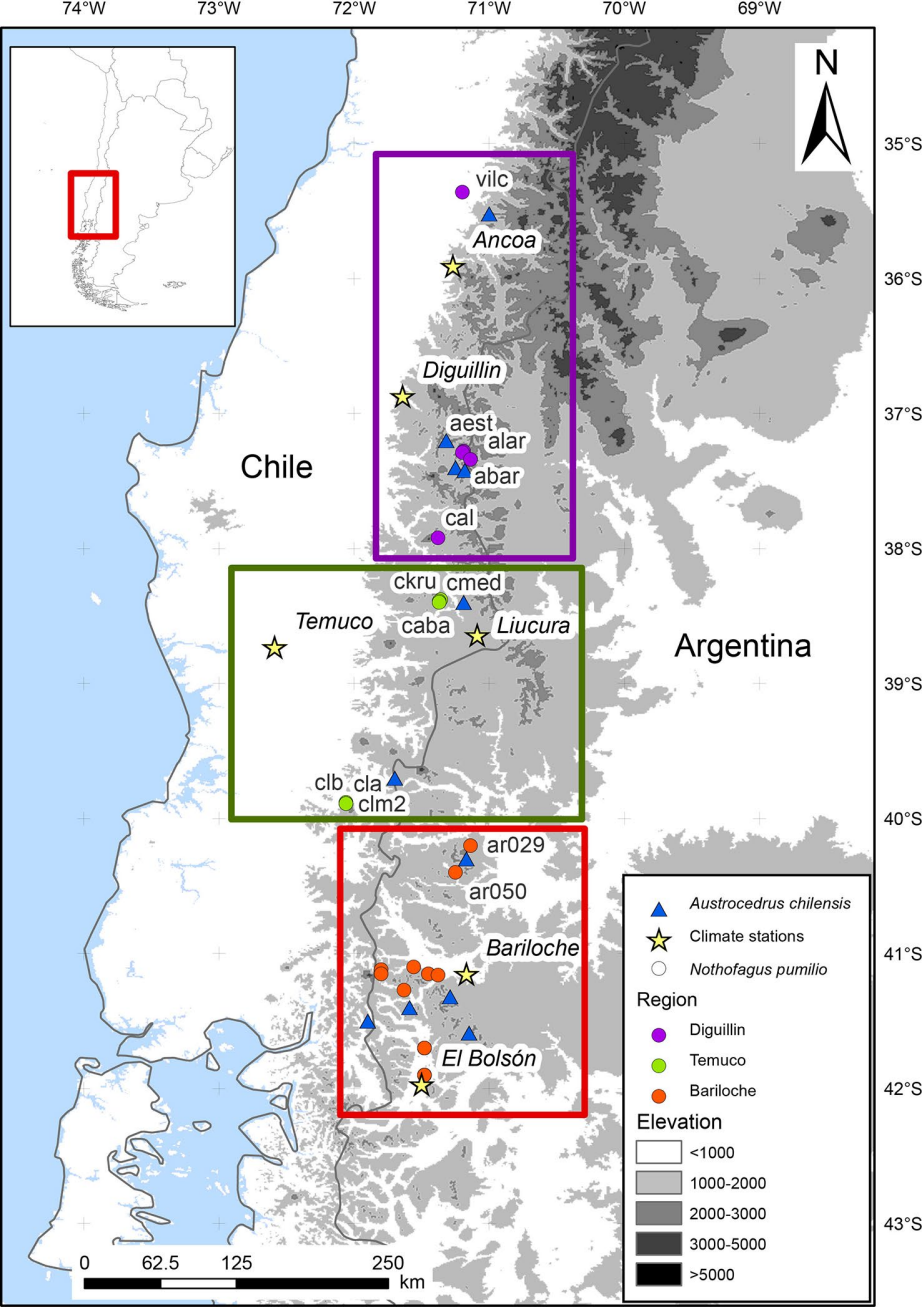
Location of the tree-ring sites and defined regions in the northern Patagonian Andes. Purple, green, and red circles indicate the *N. pumilio* stands in each region (Diguillin, Temuco, and Bariloche, respectively). Blue triangles indicate the *A. chilensi*s sites. Yellow stars indicate the two selected meteorological stations of each region. Grey scales illustrate the elevation range. Site codes are shown in **Supplementary Table S1**.

We used an extensive tree-ring network covering most of the northern *N. pumilio* distribution. It comprises tree-ring samples from 30 N*. pumilio* stands (777 trees and 1255 cores; **Figure 1**; **Supplementary Table S1**). Tree-ring records from 16 stands were retrieved from the International Tree-Ring Databank (ITRDB). Additionally, we also utilized tree-ring samples from 11 sites of *A. chilensis* (**Supplementary Table S2**; **Figure 1**).

The wood cores were air dried, mounted on wooden supports, and progressively sanded until rings were perfectly visible. Afterward, tree cores were visually cross-dated by identifying key interannual growth patterns and measured using a semi-automatic device (Velmex Inc., USA) with a 0.01 mm resolution. Finally, the cross-dating process was statistically confirmed using the program COFECHA (Holmes, 1983). We used the Southern Hemisphere tree-ring dating convention, which assigns an annual ring to the calendar year in which radial growth begins (Schulman, 1956).

To remove age-related effects, we detrended the tree-ring width series using a two-step procedure. We fitted negative exponential and 50-year long splines, which maximize the high-frequency climatic information and minimize the non-climatic variance related to ontogenetic trends and local disturbances (Helama et al., 2004). We also applied autoregressive modeling to remove tree-ring width series autocorrelation. The resulting residual indices from each site were averaged using a robust biweight mean to obtain a chronology of tree-ring width indices (TRWi). The detrending process was carried out using the packages *dplR* (Bunn et al., 2016) and *detrendeR* (Campelo et al., 2012) in the R statistical language (R Development Core Team, 2017). The quality of the tree-ring chronologies was assessed with several dendrochronological statistics (**Supplementary Tables S1** and **S2**; Fritts, 2001), namely mean sensitivity (MS), first-order autocorrelation of raw width data (AC1), mean correlation between trees (rbt), mean correlation among tree-ring indices (rbar) and Expressed Population Signal (EPS), which measures the intra-chronology signal variability compared with a perfect infinitely replicated chronology (Wigley et al., 1984).

### Identification of Homogenous Regions and Climate Data

To perform further analyses, we did a preliminary analysis to identify *N. pumilio* homogeneous regions considering tree growth patterns and climatic sensitivity. To do it, we performed two Principal Component Analyses (PCA), one on the residual chronologies (**Supplementary Figure S1A**) and a second one on the growth-climate relationships (**Supplementary Figure S1B**) for the common period 1900 to 1991 (**Figure 1**). Three regions were established considering the relative position in the PCA analysis: Diguillin (Chilean north distribution), Temuco (Chilean west distribution) and Bariloche (Argentinean east distribution). Temuco and Bariloche showed larger similarities; however, we split them into two regions due to differences in the thermal amplitude related to distance from the Pacific ocean (**Figure 1**). We selected two meteorological stations per region, as close as possible to the sampling sites (**Supplementary Table S3**). Daily absolute minimum and mean temperatures were standardized to have a mean of zero and a standard deviation of one (z-scores). For standardization, we used the mean and the standard deviation of the 1987 to 2013 common period. After that, we constructed a regional mean integrating the two meteorological stations in each region (**Supplementary Figure S2** and **Table S3**). We analyzed the period with precise climatic information for every region, from 1965 to 2016 for Diguillin, from 1950 to 2018 for Temuco, and from 1939 to 2018 for Bariloche (**Supplementary Table S1**). In Bariloche, missing meteorological information occurred in the 1950s, early 1960s, and 1970s (**Supplementary Figure S2**), periods that were excluded from analysis.

### Criteria for Detecting Past Spring Frosts

**Figure 2:**
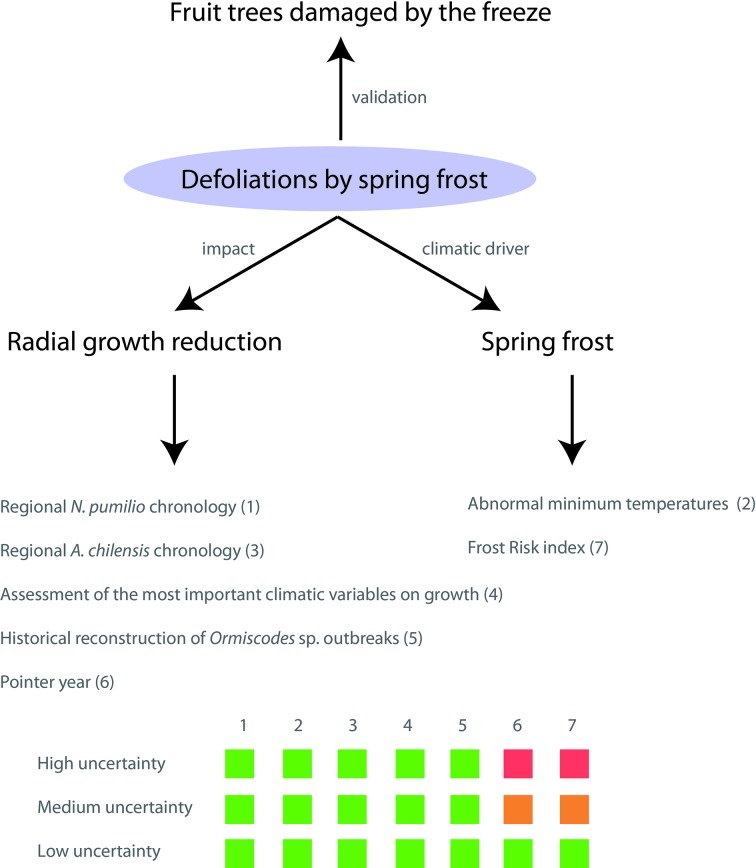
Conceptual framework for identifying years with frost damages in *N. pumilio*. The confidence levels (low, medium, and high uncertainty) were displayed by squares of three colours. Green indicates the necessity to meet those criteria. Orange indicates that one out of the two additional criteria should be met. Red indicates that this criterion is not required. For more details on different methods see the corresponding section below

We used multiple, complementary, and independent tree ring and climatic methods to develop a conservative approach to detecting past damages caused by spring frosts (**Figure 2**). We considered the following five criteria: *i)* spring frosts induce significant local growth reductions in comparison with the mean regional tree-ring chronology, *ii)* frost damage is concurrent with anomalies in the absolute minimum temperatures during the leaf unfolding period, *iii)* the evergreen *A. chilensis* does not show significant growth reductions for these years, *iv)* during years with spring frost damage other important climatic variables that could also explain the growth reductions do not show atypical values, *v)* there are no reconstructed insect outbreaks for these years at the sampled stands. Finally, we incorporate two additional quantitative criteria: *i)* the growth reductions are negative pointer years, present in 50% of tree-ring series at a site, and *ii)* in years with potential foliage damage there is a high “frost risk” index, which is defined below. These two criteria were used to reduce the uncertainty level of the reconstruction, but were not considered eliminatory. Finally, reconstructed frost events were validated by records of frost damage in agricultural plantations, mainly of apple and pear trees, in Alto Valle, Rio Negro, Argentina. Below, we explain each criterion in detail.

#### Criterion 1. Detection of Local Growth Reductions

We identified local growth reductions in relation to the mean regional growth chronology, i.e., low radial growth in a certain year and site with respect to the mean regional growth chronology in that year. We generated an individual regional *N. pumilio* chronology for each region using site chronologies within each region (**Supplementary Figure S1**; **Table S1**; Fritts 2001). We calculated the difference between each site chronology and the mean regional growth index excluding the target site. We identified significant local growth reductions when the site chronology in a certain year was 1.5 SD below the mean regional chronology in that year (period 1950–1991).

#### Criterion 2. Daily Minimum Temperatures During Leaf Unfolding

We analyzed the daily minimum temperatures in the period between the beginning of October and the first fortnight of November, when leaf unfolding of *N. pumilio* usually occurs (Rush, 1993; Martínez Pastur et al., 2007). We selected those years with minimum absolute temperatures below 1.5 SD (z-scores lower than −1.5; **Supplementary Figure S2**). We do not use a minimum temperature threshold since the critical temperature threshold may vary considerably in each region depending on the location of the meteorological stations.

#### Criterion 3. Comparison With ***A. chilensis*** Regional Chronology

*A. chilensis* growth is considered to be very sensitive to drought stress (Villalba and Veblen, 1998; Mundo et al., 2010; Villalba and Veblen, 1997). Despite the growth response to climate not being identical to that of *N. pumilio* (Paritsis et al., 2009), we used *A. chilensis* tree-ring chronologies as an additional criterion to identify the occurrence of droughts or other natural disturbance (e.g., volcanoes, earthquakes or forest fires).

Years with local growth reductions (*Criterion 1*), were compared to the regional *A. chilensis* chronology (**Table S2**). If *A. chilensis* chronology showed a growth reduction below 1.5 SD (1950–2003 period) in that year, we attributed the growth reduction to either drought stress or non-climatic common constraints (e.g., volcanoes, earthquakes or forest fires; Kitberger et al., 1995). If an unusual growth reduction in the *A. chilensis* chronology did not occur, then we assumed that it could be due to damage caused by low temperatures in spring.

#### Criterion 4. No Deviations in Other Important Climate Variables for Growth

Climate variability is the major factor determining tree growth, and therefore, we analyzed if monthly precipitation and temperature anomalies could also explain the local growth reductions. First, we related the tree-ring chronologies to monthly climate data using Pearson correlation coefficients (Fritts, 2001). We used long-term monthly mean temperature and precipitation from the Climate Research Unit (CRU) TS 1.6.9. database (Mitchell and Jones 2005). We performed the analysis from March of the current growing season back to January of the previous growing season. Second, in each site, we selected the climatic variables that significantly correlated with tree growth (*P* < 0.01). Where no climatic variable showed significant correlations with growth, the variables that most frequently influences regional growth was considered (e.g., November and December temperature and precipitation in Bariloche region). Finally, for each site, we selected years without deviations in the most important climatic variables for growth (1 SD for the period 1950–2003).

#### Criterion 5. No Documented or Reconstructed Biotic Agents Damages

Insect outbreaks are scarce in the *N. pumilio* northern distribution area, however *Ormiscodes* genus (Lepidoptera: Saturniidae) outbreaks do occur (Paritsis and Veblen, 2011). Since both frost damage and insect outbreaks cause narrow rings, we discarded frost damages in those years with documented or reconstructed outbreaks in northern *N. pumilio* forests, such as 1986 and 2001 (Paritsis and Veblen, 2011).

#### Additional Criterion 1. Pointer Years

Spring frost damage causes sharp and punctual tree-ring growth reductions, which were identified at the population level as negative pointer years. We defined a pointer year when more than 50% of the series in a site showed a negative normalized growth deviation greater than the 0.5 threshold on the so-called Cropper values (Cropper 1979) over a 5-year period. We used the *pointer.norm* function of the package *pointRes* (van der Maaten-Theunissen et al., 2015) in R environment (R core Team 2017). This criterion was not considered as eliminatory since our approach is focused on the mean tree-ring chronology at stand level. Spring frost events with partial damage, only affecting trees with early leaf phenology, could induce a significant local growth reduction but not so generalized to be identified as a pointer year. However, we considered the additional fulfilment of this criterion as an indicator of very intense damage events.

#### Additional Criterion 2. Temperature Variability Before and During the Leaf Unfolding

The sequence of temperatures during the leaf development is critical for the occurrence of leaf damage by freezing temperatures (Augspurger 2009 Augspurger, 2013). Abnormally warm temperatures in early spring accelerate leaf flush and expose vulnerable phenophases to frost risk. We developed an index to cover the thermal amplitude in this period as a proxy of frost risk (FR), which we defined as:

(1) $$\mathrm{FR}=\mathrm{Tmea}n_{\mathrm{sep}(2)-\mathrm{oct}(1)}-\mathrm{Tmi}n_{\mathrm{oct}-\mathrm{nov}(1)}$$

where *Tmean* *_sep(2)-oct(1)_* is the mean temperature before or during leaf unfolding, from September 15 and October 15, and *Tmin_oct-nov(1)_* is the absolute minimum temperature in the period when leaf unfolding usually starts (October and the first fortnight of November; Rush 1993;Martínez Pastur et al., 2007). We selected years with high values of the frost risk index (> 1.5 SD; **Supplementary Figure S2**). The selected period is relatively wide to cover the effect of elevation on leaf unfolding phenology.

We did not consider this criterion as eliminatory, because frost damage can also occur without previous warm early springs; however this criterion helped to reduce uncertainty.

### Criteria Integration

We integrated all these criteria to determine a four level confidence scale (**Figure 2**): *i) high uncertainty*: a local growth reduction occurred simultaneously with low minimum temperatures during leaf unfolding. However, growth reduction was not so important as to meet the requirements of pointer years and frost risk index was not high; *ii)* *medium uncertainty*: a local growth reduction occurred simultaneously with unusually low minimum temperatures, and either it was a negative pointer year or showed a high frost risk index; *iii) low uncertainty*: all the tree-ring and climatic criteria were met; *iv) validated*: all the criteria were met, and frost damage was validated with frost records of agricultural crops (1980 and 1992 in Bariloche; Tassara, 2007). Owing to the nature of spring frosts, they could also coincide with other climatic constraints (e.g., drought) in the same growing season, thus we also indicated when other climatic constraints may have contributed to any recorded growth reduction.

In years with low uncertainty or validated damage, we analyzed the daily sequence of minimum and mean temperatures between October and November to pinpoint the day of year (DOY) when the critical frost probably occurred. For this purpose, we determined the day when the difference between the mean temperature from the previous 10 days and the absolute minimum temperature for that day was the largest. Warm periods followed by extreme frosts showed the highest values of frost risk index.

## Results

### Identified Regions and Growth Responses to Climate

The three regions differed in year-to-year growth variability as well as growth-climate relationships (**Supplementary Figure S1**; **Figure 1**). Nevertheless, the differences between the Chilean west (Temuco), and the Argentinean east (Bariloche) regions were less evident.

**Figure 3:**
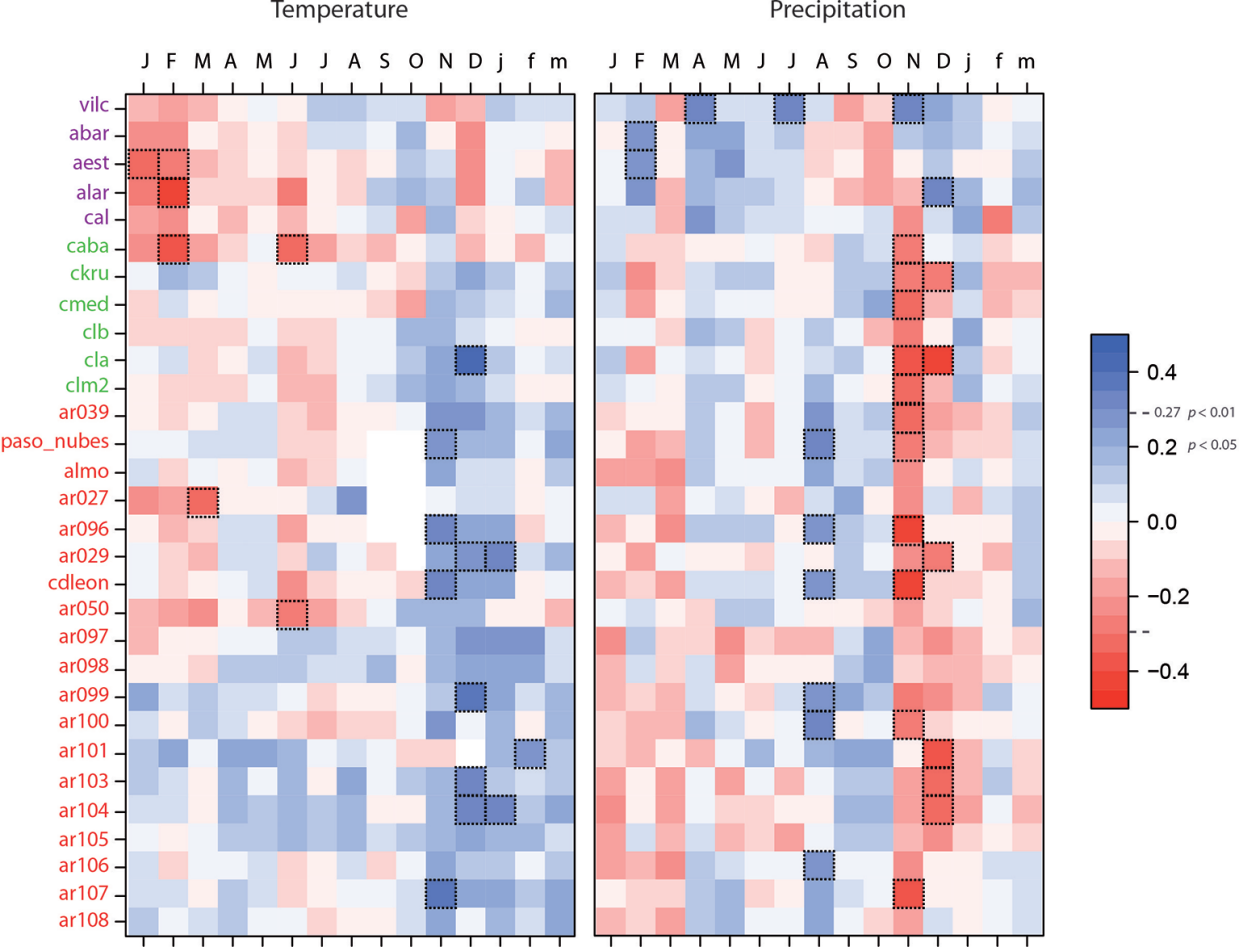
Growth-climate relationships for the studied *Nothofagus pumilio* populations. Correlations were performed across the common period 1900–1991. Sites are sorted by latitude. Purple sites corresponded to Diguillin region (Chilean north distribution), green sites to Temuco region (Chilean west distribution) and red sites to Bariloche region (Argentinean east distribution). Correlations showing *P* 0.01 are highlighted with dotted line boxes.

Tree growth in the Chilean-northern sites (Diguillin) was enhanced by wet (February) and constrained by warm (Jan-Feb) conditions during the previous summer (**Figure 3**). In the western Chilean sites (Temuco), tree growth was favored by warm springs (November and December), whereas abundant rains during this period, which indirectly lower temperatures, negatively influenced tree growth. Finally, in the Argentinean east region (Bariloche), growth was favored by wet late winters (August) and warm spring and early summer conditions (November, December, and January). As in the western region of Chile, dry and warm springs (November and December) favored tree growth. These climate-growth relationships served as a basis to select the most relevant climatic variables controlling growth (**Figure 3**).

### Spring Frost Damage Years Identification

**Figure 4:**
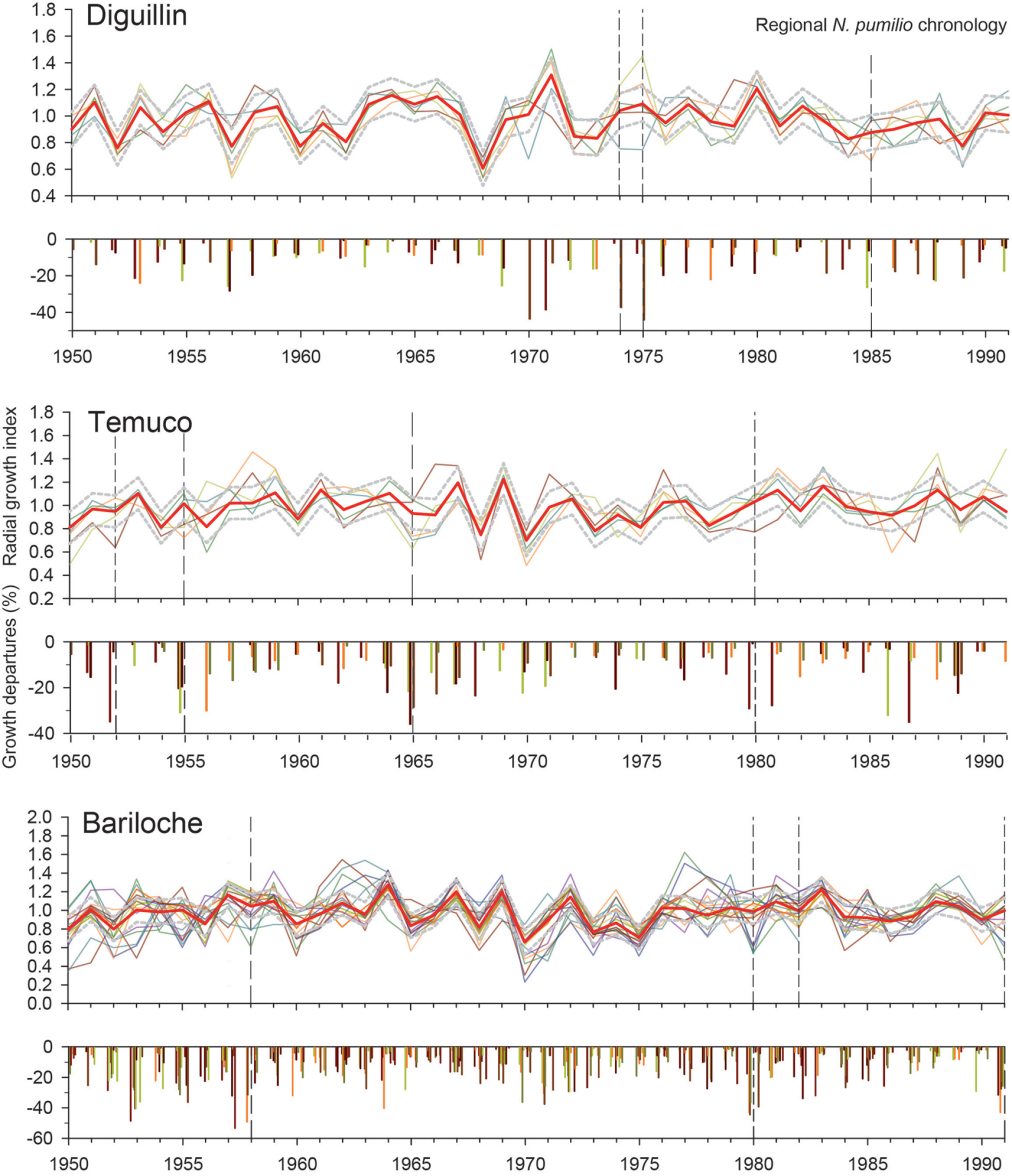
Radial growth index in each stand (fine lines) and local growth reductions (vertical bars) for the three defined regions (Diguillin, Chilean north distribution; Temuco, Chilean west distribution, and Bariloche, Argentinean east distribution) related to the regional mean chronology (red lines). The upper and lower dotted grey lines indicate the SD limits. Vertical dotted lines indicate the reconstructed frost damage events with different uncertainty levels (see **Table 1**). Note that for this figure the regional chronology was performed using the set of all chronologies, while in the analysis the series from the target site were excluded to avoid redundant information

We identified several years and sites with significant local growth reductions and low minimum temperatures in spring. These potential years of spring frost damage were more frequent in Bariloche, where low minimum temperatures and growth reductions were mainly coincident in 1958, 1980, 1982, and 1991 (**Figure 4**). In Temuco, we detected local growth reductions and low spring minimum temperatures in 1952, 1955, 1965, and 1980, while in Diguillin, mainly occurred in 1974, 1975, and 1985. Some tree-ring sites showed local growth reductions in the absence of critical spring freezing temperatures. For example, growth reductions at the “ckru” site in 1986 and at the “caba” site in 1987 were both associated with deviations in other important climatic variables for growth (**Figure 3**). In addition, a sharp growth reduction was observed at the “cal” site in the Diguillin region, which was linked to recently tree dieback.

**Figure 5:**
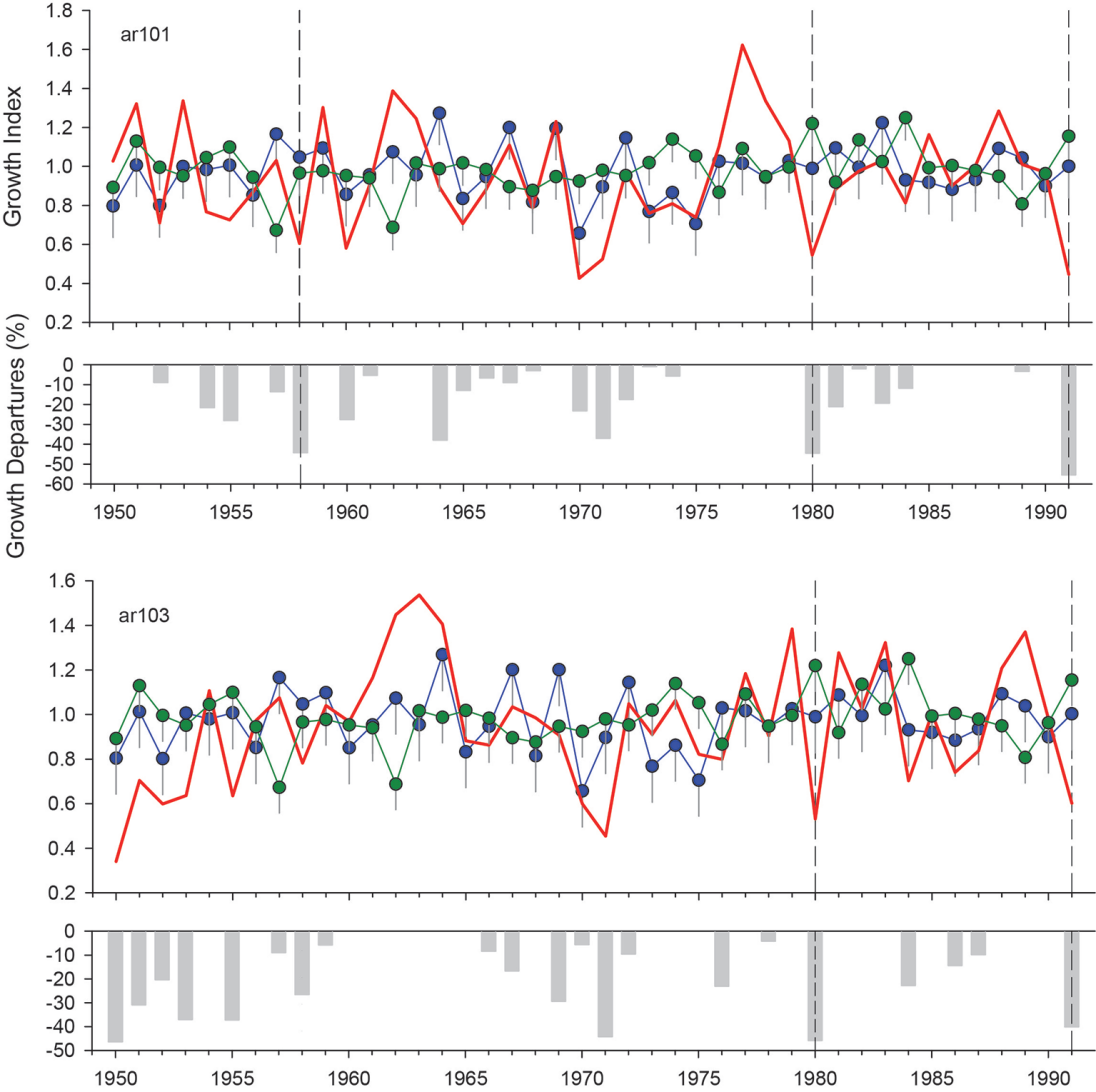
Selection of potential *N. pumilio* growth reductions by spring frosts considering the local (red lines), regional (blue lines) and *A. chilensis* (green lines) chronology. The vertical lines indicate the standard error. The lower bar graphic indicates the growth departures (%) from the regional *N. pumilio* chronology. Vertical grey discontinuous lines show sharp growth reductions not present in the regional mean or the *A. chilensis* chronology, and with significant minimum temperatures during the leaf unfolding.

We illustrate our methodological approach with an example. The ar101 and ar103 sites (both in the Bariloche region) showed local growth reductions in 1958, 1980, and 1991 (the latter for ar101 only; **Figure 5**). In 1958 and 1980, no other climatic constraints on growth were identified (November and December; **Figure 3**) nor were there lower growth rates in the *A. chilensis* chronology (**Figure 5**). Additionally, these years showed extreme minimum temperatures during leaf unfolding period (**Supplementary Figure S2**). Finally, 1980 showed a high frost risk index and documented records of frost damage in fruit trees.

By integrating all the criteria, we were able to reconstruct two spring frost events at four stands in the Bariloche region occurring in 1992 and especially 1980 that were validated with agricultural information (**Table 1**). Moreover, we recorded an event with low uncertainty in 1957, but with low growth rates in the *A. chilensis* chronology, which probably indicates an additional drought stress. We also detected three medium-uncertainty events in 1958, 1982, and 1991, when some forests received abundant December precipitation that might have influenced negatively on growth. Finally, two events with high uncertainty were registered in 1958 and 2010. In the Temuco region, we identified different potential frost events with low and medium uncertainties in 1952, 1955, 1965, and 1980, although with the exception of 1980 all those years also showed other local climatic constraints (**Table 1**). Finally, in the Diguillin region, we detected several episodes in 1974, 1975, 1978, 1985, and 1995, although only 1974 with low uncertainty and 1975 with medium uncertainty, were not associated with other climatic constraints (**Table 1**).

**Table 1:** Reconstructed spring frost events and uncertainty levels based on the different criteria (see section Criteria for Detecting Past Spring Frosts). The values in brackets indicate the minimum daily temperature recorded in that year in the two meteorological stations considered for each region during leaf unfolding. Deviations of any of the important climatic variables on growth are also indicated, as well as lower growth rates in the *A. chilensis* chronology (EG). Site codes are shown in **Supplementary Table S1**.

Region	Site	Reconstructed spring frosts (°C; Other drivers)			
		Validated	Low Uncertainty	Medium Uncertainty	High Uncertainty
Diguillin	vilc				1995 (−2/0.2; EG)
	abar				1985 (−1/0; PFeb)
	aest				
	alar			1978 (−3/1; EG)	
	cal		1974 (−1/−3)	1975 (−4/−1.5)	
Temuco	caba			1980 (−1.2), 1952 (−1; high TFeb)	
	ckru			1955 (−1; high PDec)	
	cmed		1965 (−1.3; high PNov)		
	clb				
	cla		1965 (−1.3; high PNov)		
	clm2				
Bariloche	ar039				
	paso_nubes	1992 (-8),			2010 (−5)
	almo				
	ar027			1982 (−6), 1957 (−9; EG)	
	ar096				
	ar029	1980 (−6)			
	cdleon				1958 (−6)
	ar050		1957 (−9; EG)		
	ar097				
	ar098			1991 (−6)	
	ar099				
	ar100			1982 (−6)	
	ar101	1980 (−6),		1958(−6), 1991 (−6; high PDic)	
	ar103	1980 (−6)		1991 (−6; high PDic)	
	ar104	1980 (−6)			
	ar105			1991 (−6)	
	ar106				
	ar107				
	ar108				

In the Bariloche region, several identified frost events showed temperatures below −5°C to −8°C in the reference meteorological station during the leaf unfolding (**Table 1**). On the contrary, temperatures in the Temuco and Diguillin regions were higher, indicating possible damages at just −1°C. However, due to the larger distances and elevation differences between the meteorological stations and the studied stands, the minimum temperatures in the forest might have been much lower.

### Thermal Characterization of Validated Past Damages

**Figure 6:**
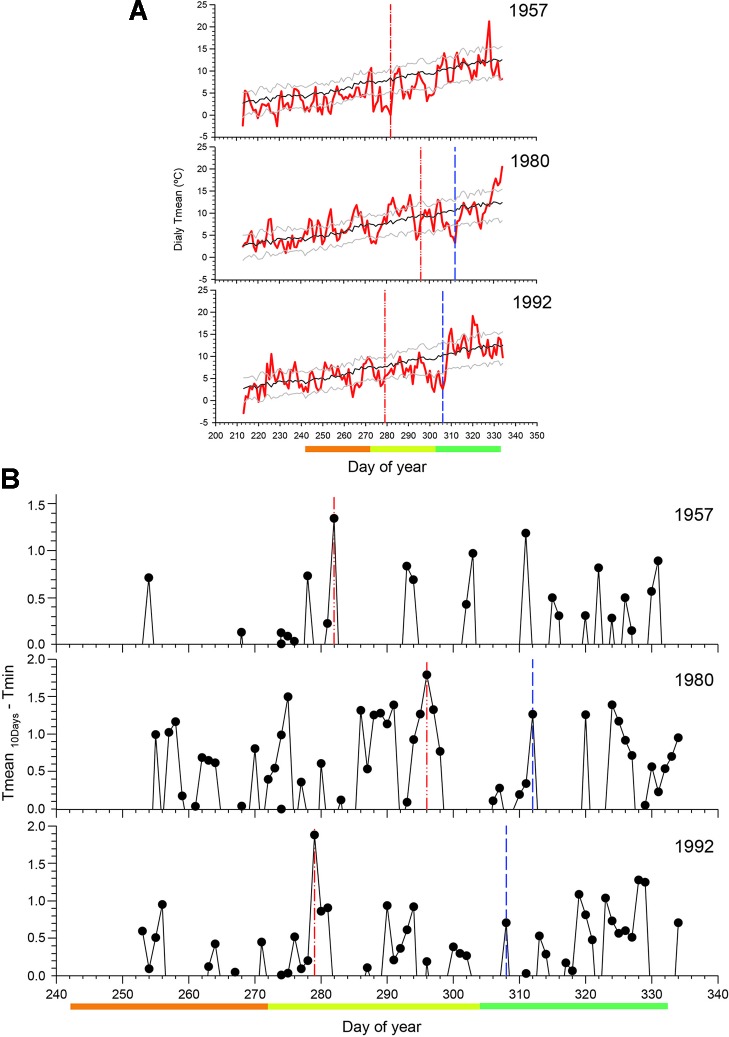
**(A)** Daily mean temperature in three selected years with low uncertainty (1957) or validated (1980 and 1992) spring frosts in the Bariloche region. The black line shows the mean in the reference period, and the grey lines the standard deviation. **(B)** Mean temperature in the previous 10 days minus the absolute minimum temperatures in that day. High values of this index indicate warm temperatures followed by extreme minimum temperatures (below zero), and thus a high probability of frost event occurrence (red line). The blue line indicates the known date of frost damage in agricultural records. The orange, yellow, and green bars indicate September, October, and November, respectively

We analyzed the daily climatic patterns in 1957, 1980, and 1992, 3 years with probable spring frost damage occurrence, with 1980 and 1992 both validated by records of severe frost damage in fruit tree plantations (**Figure 6**). We detected higher thermal amplitude, as reflected by the frost risk index, at the beginning of October 1957 (DOY 282) and 1992 (DOY 279), while in 1980 the highest amplitude occurred at the end of October (DOY 296). These 3 years showed warm temperatures before the critical frost occurrence (**Figure 6A**). This phenomenon was particularly clear in 1980 with warm temperatures during 8 days in the middle of October followed by extremely low temperature of −6°C in late October. On the other hand, we found a mismatch between the date when fruit trees damage was documented in early November (DOY 312 in 1980, DOY 308 in 1992) and the day with the highest thermal amplitude that induced the frost and caused damages in *N. pumilio* (**Figure 6B**).

## Discussion

To our knowledge, this study represents the first attempt to reconstruct foliage damage occurrence by spring frosts in a deciduous tree species in South America, and thus a new step towards understanding the role of spring frosts in the dynamics of deciduous forests. Our findings support the initial hypothesis that these extreme climatic events are not only restricted to the Northern Hemisphere. We used multiple pieces of evidence to assess the potential incidence of spring frosts on *N. pumilio* forests, specifying four confidence levels as a function of the fulfilment of increasingly restrictive criteria. First, we identified local growth reductions not induced by regional or local climatic constraints or other natural disturbances (e.g., insect outbreaks, volcanoes, forest fires or earthquakes), but rather by abnormally freezing temperatures in spring, that in some cases matched documented frost damages in orchards (**Figure 4** and **Table 1**). Second, late frosts damaging *N. pumilio* foliage were shown to be the result of a combination of abnormally warm early springs, promoting earlier leaf flushing, followed by critically low minimum temperatures in this stage (Augspurger 2009; Augspurger, 2013) (**Figure 6**). The geographical pattern of spring frost damages was heterogeneous: the eastern populations of *N. pumilio* on the Argentinean side of the Andes showed a higher incidence of spring frosts, reflecting a more continental climate (**Table 1**). Hence, deciduous broadleaf forests worldwide under more continental climates, e.g., far away from oceanic influence, may be more sensitive to frost damage. This combination of dendroecological and climatic information provides a conservative approach to identifying foliage damage caused by spring frosts.

Growth-climate relationships evidenced regional differences in the sensitivity of *N. pumilio* growth to climate, especially between northern and southern populations (**Figure 3**). Climatic conditions affecting tree growth shift from drier conditions in northern sites in Chile, to wetter and cooler conditions in the high-elevation forests in the southern sites (Villalba et al., 1997; Lara et al., 2001; Lara et al., 2005; Daniels and Veblen 2004; Álvarez et al., 2015; Lavergne et al., 2015). Above-average spring temperatures limit tree growth in the northern forests under a Mediterranean climate (Lara et al., 2001), whereas the southern sites share common climatic limitations in wet and cool sites (Villalba et al., 1997; Lara et al., 2005). In the southern sites, the negative correlation of tree growth with the November and December precipitation (**Figure 3**) has been linked with the shorter growing season due to larger cloud cover and prolong snow in late spring (Lara et al., 2001; Lara et al., 2005; Lavergne et al., 2015). Therefore, northern populations are mainly limited by precipitation during the early growing season, while tree growth in the southern populations is mostly controlled by temperature (Villalba et al., 1997; Lara et al., 2001; Álvarez et al., 2015; Lavergne et al., 2015).

The *A. chilensis* records provided a control for the effects of major climatic and non-climatic disturbances. Tree-growth responses to climate showed some differences between *A. chilensis* and *N. pumilio* (Paritsis et al., 2009), partially due to the absence of site-level paired *N. pumilo-A. chilensis* chronologies. However, *A. chilensis* is the only conifer that spatially accompanies the 30 selected stands of *N. pumilio* located along the 750 km transect. In spite of this limitation, *A. chilensis* provided relevant information; in the Bariloche region all the proposed criteria suggested a frost damage event in 1957, but the *A. chilensis* chronology also showed a significant growth reduction. Therefore, it seems probable that an additional negative constraint could have affected both species.

Insect outbreaks could be confused with spring frost events. Fortunately, the existence of a reconstruction of insect outbreaks in the northern *N. pumilio* distribution area (Paritsis et al., 2009; Paritsis and Veblen, 2011) reduced this uncertainty. These authors also applied multiple and complementary tree-ring methods to reconstruct *Ormiscodes* outbreaks for the 1850 to 2005 period. Their results indicate that insect outbreaks are relatively rare in northern Patagonia, with an incidence of 1.5 outbreaks per century at stand level, although their frequency is expected to increase in the 21th century (Paritsis and Veblen, 2011). We could not fully discard that the analyzed forests may have been affected by some past insect outbreak not previously documented (e.g., 1970 in “caba” site, Diguillin region). However our proposed methodology is robust enough to avoid misidentifying insect defoliation as frost damage. Although insect outbreaks are associated with dry and warm spring conditions (Paritsis and Veblen, 2011; Paritsis and Veblen 2010), severe frosts during the early caterpillar development could be also extremely harmful for development of an abundant population of defoliating insects. Thus, the climatic trends associated with an increased risk of spring frost occurrence, are not conducive to major insect outbreaks.

Intra-annual thermal variations were quite similar in 1980 and 1992 (**Figure 5**), the 2 years when foliage damage by frost were validated by damage in orchards (Tassara, 2007), but also in 1957, a year with low uncertainty of frost damage occurrence. Warmer temperatures, for 2 to 8 days coincident with leaf unfolding period, were followed by a sharp drop in temperature (−6°C to −9°C in the reference meteorological station). Warmer temperatures promote an earlier initiation of the sprouting phenophase, increasing frost vulnerability (Augspurger 2009; Augspurger, 2013; Hufkens et al., 2012; Menzel et al., 2015; Príncipe et al., 2017). Although the distance between orchards and the studied stands is large (ca. 300 km), we consider this information as an additional indicator of enough quality to validate our frost damage reconstruction in *N. pumilio*.

During the 2 years (1980 and 1992) with damage recorded in orchards, we diagnosed a possible mismatch between the date when we found the maximum thermal amplitude and the date of the agricultural damages. We have two potential explanations. First, the agricultural damages occurred mainly in the fruits (Tassara, 2007), which are likely to occur in a different time window to damage in the foliage of *N. pumilio*. Certainly, the phenological timing of fruit development in cultivated trees on the Argentinean plains differs from that of leaf flushing of *N. pumilio* at higher elevations, creating dissimilar vulnerability periods. Second, we also hypothesized that the damages could have occurred simultaneously during the first fortnight of November, since we also found a high thermal amplitude index at this time. In this scenario, it is assumed that no damage resulted from the first frost that occurred in late October due to since the buds had not yet sprouted. Under either of these two explanations, frost damage occurrence in these years was especially harmful owing due to the preconditioning high temperature oscillation in a short period and the very low temperatures coincident with leaf unfolding.

We detected frost damage in 1980 in several studied areas, with four sites displaying important growth reductions in the Argentinean sites, but also one site (“caba”) showing medium uncertainty in the Temuco region in Chile (**Table 1**). The factors promoting late frost were more noticeable in 1980, when a prolonged 12 days warm period was followed by −6°C minimum temperatures in late October (**Figure 6B**). This long warm period could have significantly accelerated leaf sprouting, leaving large forest areas predisposed to freezing temperature damages.

Temperature thresholds for frost damage have been established at −7°C for widespread damage in Eastern US (Gu et al., 2008), and −3°C (Dittmar et al., 2006; Príncipe et al., 2017) or −5°C (Menzel et al., 2015) in European *F. sylvatica* forests. Our thresholds varied depending on *N. pumilio* forest location (**Table 1**). We reported frost damage with temperature data from meteorological stations ranging from −1°C in the Chilean regions to −6°C in the high-elevation Argentinean forests. However, temperatures in the forests could have been significantly lower than in the meteorological stations, especially in the western sites due to differences in elevation between the stations and forests.

Hadad et al. (2019) have recently reconstructed spring frosts in northern Patagonia based on annual anatomical damage in the xylem (“frost rings”) of the conifer *Auraucaria araucana* for the period AD 1256 to 2008, which remarks the broad incidence of spring frost on different tree species in the region. The authors pointed out several large-geographical freezing events mainly related to “La Niña” conditions in the 20th century, such as 1916, 1941, and 1948. Frost rings of *Auraucaria araucana* are not related to growth reductions, as is the case in *A. chilensis* (Muñoz-Salazar et al. in prep.). Owing to the lack of daily climatic data in these years, we could not evaluate the possible incidence on *N. pumilio*, although in general we observed a sharp growth reduction in 1941, but not in 1916 or 1948. Nevertheless, the timing of xylogenesis and leaf flushing differs between these species, creating differences in the period of frost damage risk between them.

Several studies have used tree rings to reconstruct spring frosts in *Fagus sylvatica* (e.g., Príncipe et al., 2017; Dittmar et al., 2006). These studies combined modeled or observed information of leaf unfolding timing with high-resolution climate data. This approach is optimal when leaf phenology can be modeled. However, no information is available for remote forests such as those in Patagonia. Moreover, an alternative approach using remote sensing may be limited by the high cloud cover and the limited spatio-temporal resolution in satellite images. Therefore, the inference of spring frost events on *N. pumilio* using our conservative approach of seven different and independent criteria are a sound alternative. We only identified years with clear evidence of severe frost occurrence and supported by the use of multiple confidence levels. The restrictive nature of our methodology may not have been effective in detecting some years when partial damage occurred (e.g., Dittmar et al., 2006; Augspurger 2009; Menzel et al., 2015).

Intrapopulation response differences to frost damage were not considered in our stand-based approach. For future dendroecological research, we recommend exploring the growth response using individual nonlinear models (Montoro Girona et al., 2017) and including, ideally, other important factors such as elevation, tree-to-tree competition or distance to forest edge. In addition, further studies about new indicators and proxies of frost damages are also needed to reconstruct the spatio-temporal pattern of these events, especially in pre-instrumental periods. In this sense, the analysis of wood traits complementary to ring width (i.e. anatomy, microdensity or isotopes) could provide new key criteria to confidently identify the incidence of these extreme events at tree level.

To conclude, the dissimilar occurrence of spring late frosts in *N. pumilio* at its northern range of distribution in the Patagonian Andes, indicates a larger incidence in the more continental Argentinean forests. The Patagonian Andes have experienced a significant warming through the 20th century, more intensely in the southern (+0.86°C) than the northern (+0.53°C) areas (Villalba et al., 2003). Importantly, climate models indicate that these trends will continue (Carril et al., 1997; Vera et al., 2006). In this climatic context, it is probable that the *N. pumilio* leaf unfolding date will advance, potentially exposing trees to an increasing risk of frost damage.

## Data Availability Statement

The datasets generated for this study are available on request to the corresponding author.

## Author Contributions

GS-B, JO, VR and RV conceived the ideas and designed the methodology. RV and DC collected the tree-ring data. GS-B and RV analyzed the data. GS-B lead the writing with insightful assistance of all authors. All the authors contributed to the discussion, read and approved the final draft.

## Conflict of Interest

The authors declare that the research was conducted in the absence of any commercial or financial relationships that could be construed as a potential conflict of interest.
