# Supplementary material for: Detecting Nothofagus pumilio Growth Reductions Induced by Past Spring Frosts at the Northern Patagonian Andes
Source: Front Plant Sci. 2019 Oct 31;10:1413. doi: 10.3389/fpls.2019.01413 (PMC6834777; doi:10.3389/fpls.2019.01413)
Supplement: Supplementary file 1 [file DataSheet_1.pdf]

Detecting *Nothofagus pumilio* growth reductions induced by past spring frosts at the northern Patagonian Andes

Gabriel Sangüesa-Barreda[1]*, Ricardo Villalba[2], Vicente Rozas[1,3], Duncan A. Christie[3,4] and José Miguel Olano[1]

Supporting Information

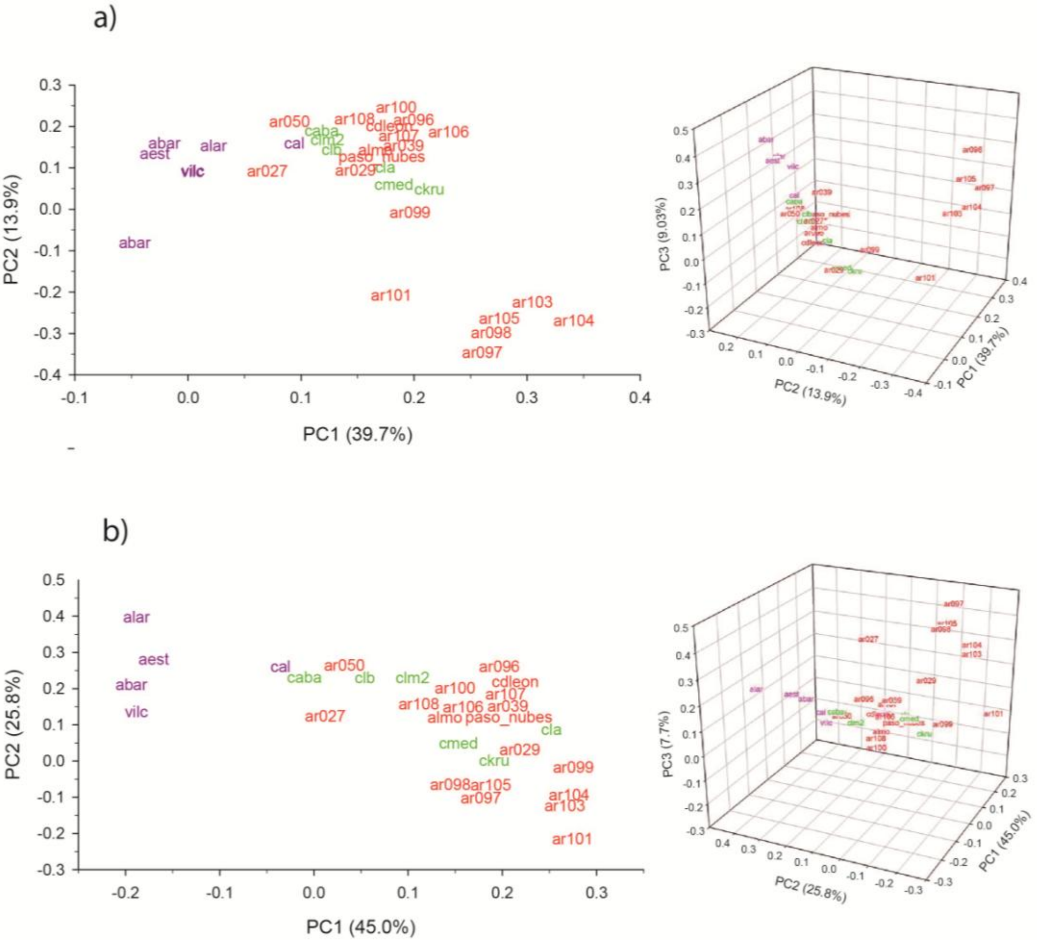

Figure S1.- Biplots and triplots of principal component analysis (PCA) of the *Nothofagus pumilio* stands (purple, green and red fonts represent sites from Duiguillin, Temuco and Bariloche regions, respectively) based on the first (PC1) and second (PC2) axes performed on TRWi for the period 1900-1991 (a), and the growth-climate relationships (b). The three bioregions were established considering the relative position of the stands in the PCA plots and their location on the western vs. eastern slopes of the Andes. We named the regions according to the main meteorological station. PCAs were conducted on the variance-covariance matrix resulting from the comparison of all TRWi chronologies and growth-climate correlations. Within-region Pearson's correlation coefficients (mean ± SE) for the period 1900-1991 were: 0.47 ± 0.06 in Diguillin, 0.49 ± 0.06 in Temuco and 0.47 ± 0.02 in Bariloche.

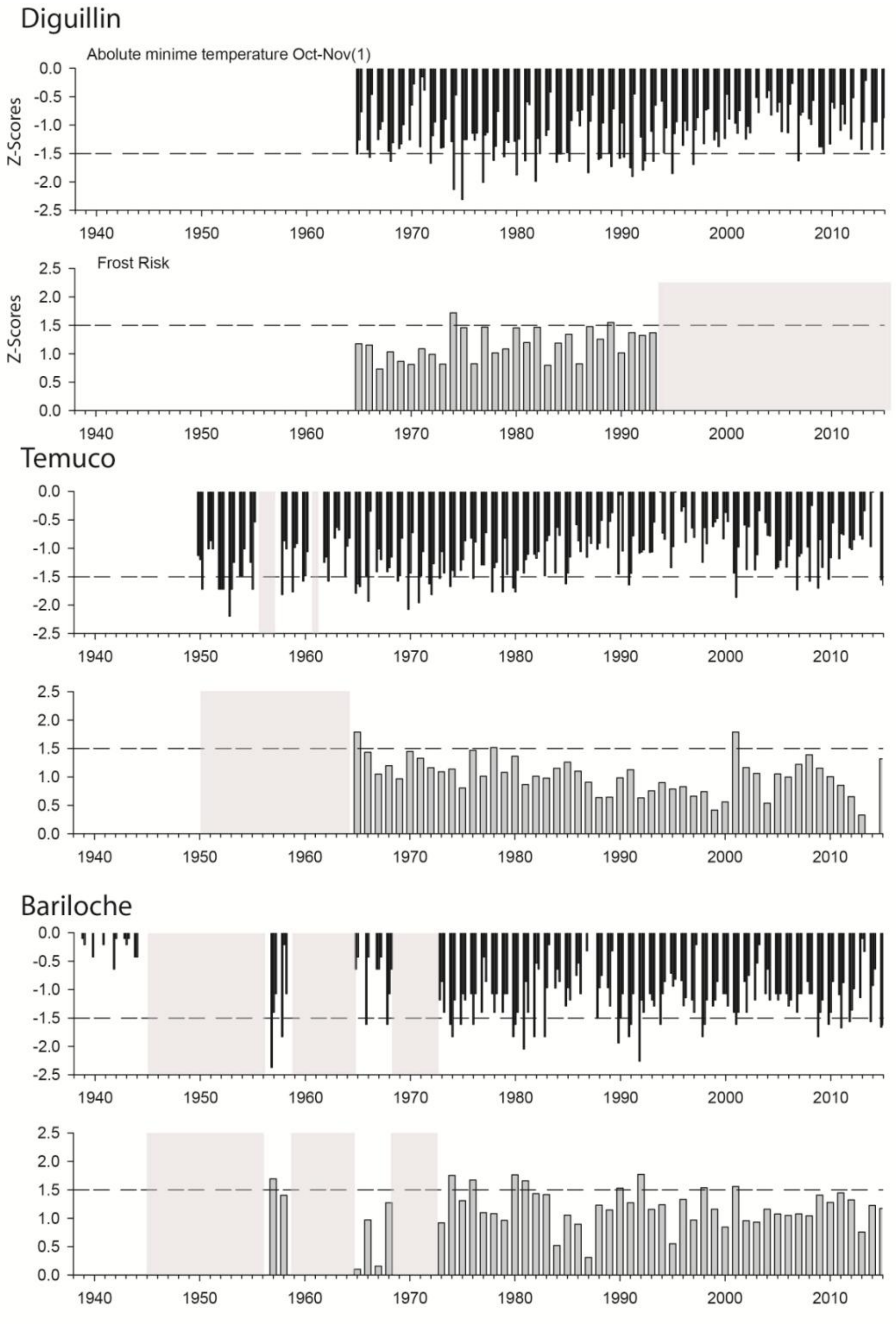

Figure S2.- Absolute minimum temperatures in October and November (first fortnight) and frost risk for the three defined regions.

Table S1.- Summary of the dendrochronological statistics of *N. pumilio* chronologies.

Site	Code	Lat (N)	Long (+E / -W)	Trees (radii)	Time span (AD)	MW (mm)	SD (mm)	MS	AC1	EPS	rbar
Vilches	vilc	-35.36	-71.20	20 (30)	1819-1996	1.42	0.70	0.33	0.53	0.88	0.27
Laja Los Barros	abar	-37.28	-71.19	32 (32)	1736-1996	1.10	0.50	0.31	0.63	0.92	0.34
Laja Las Cuevas	aest	-37.29	-71.20	17 (26)	1704-1995	1.10	0.47	0.28	0.64	0.85	0.30
Laja Lenga Larga	alar	-37.34	-71.14	23 (24)	1728-1996	1.10	0.47	0.28	0.65	0.89	0.35
Callaqui	cal	-37.92	-71.38	23 (35)	1772-2005	1.29	0.60	0.30	0.64	0.88	0.24
**Regional Chrono Diguillin**	**RM Diguillin**			**115 (147)**	**1704-2005**	**1.19**	**0.55**	**0.30**	**0.62**	**0.94**	**0.18**
Conguillío Lenga Abajo	caba	-38.38	-71.36	29 (49)	1728-1996	1.09	0.55	0.31	0.66	0.94	0.31
Conguillío krumholz	ckru	-38.39	-71.37	19 (27)	1700-1995	0.79	0.42	0.37	0.58	0.88	0.35
Conguillío Lenga media	cmed	-38.40	-71.37	25 (26)	1850-1996	0.82	0.40	0.36	0.59	0.92	0.38
Choshuenco Bajo	clb	-39.88	-72.06	89 (105)	1611-2010	1.14	0.59	0.30	0.69	0.94	0.24
Choshuenco Alto	cla	-39.89	-72.06	68 (83)	1768-2010	1.35	0.67	0.31	0.61	0.94	0.26
Choshuenco Medio	clm2	-39.89	-72.06	90 (108)	1628-2011	0.96	0.48	0.30	0.68	0.95	0.26
**Regional Chrono Temuco**	**RM Temuco**			**320 (398)**	**1611-2011**	**1.09**	**0.55**	**0.31**	**0.65**	**0.97**	**0.18**

Site	Code	Lat (N)	Long (+E / -W)	Trees (radii)	Time span (AD)	MW (mm)	SD (mm)	MS	AC1	EPS	rbar
Glaciar Frias	ar039	-41.10	-71.56	10 (21)	1595-1985	1.40	0.71	0.32	0.66	0.86	0.36
Paso Nubes	paso_nubes	-41.12	-71.80	42 (83)	1644-2011	0.88	0.49	0.29	0.70	0.93	0.23
Castaño Overo2	almo	-41.15	-71.80	64 (168)	1539-2011	0.91	0.44	0.31	0.63	0.96	0.24
Castaño Overo	ar027	-41.15	-71.45	15 (21)	1626-1982	1.42	0.67	0.27	0.72	0.80	0.25
Co Diego de León	ar096	-41.16	-71.38	19 (29)	1546-1991	0.91	0.42	0.32	0.64	0.94	0.45
Chapelco	ar029	-40.20	-71.14	15 (27)	1766-1985	0.95	0.45	0.39	0.53	0.92	0.36
Co Diego de León2	cdleon	-41.27	-71.63	51 (153)	1546-2011	0.98	0.47	0.32	0.65	0.97	0.33
Paso Córdoba	ar050	-40.40	-71.25	20 (32)	1692-1986	1.17	0.55	0.28	0.66	0.90	0.33
Diego de León	ar097	-41.70	-71.48	7 (10)	1870-1991	0.66	0.38	0.46	0.58	0.85	0.39
Paso Nubes1	ar098	-41.70	-71.48	6 (8)	1850-1991	0.97	0.55	0.45	0.60	0.82	0.44
Paso Nubes2	ar099	-41.70	-71.48	10 (18)	1718-1991	0.76	0.41	0.33	0.67	0.81	0.33
Paso Nubes3	ar100	-41.70	-71.48	12 (23)	1701-1991	0.94	0.46	0.30	0.68	0.91	0.36
Paso Nubes4	ar101	-41.70	-71.48	6 (7)	1861-1991	0.47	0.37	0.44	0.63	0.61	0.23
Castaño Overo1	ar103	-41.90	-71.48	6 (9)	1808-1991	0.42	0.25	0.40	0.67	0.85	0.45
Castaño Overo3	ar104	-41.90	-71.48	9 (14)	1820-1991	0.46	0.28	0.46	0.58	0.90	0.45
Castaño Overo4	ar105	-41.90	-71.48	10 (13)	1820-1991	0.67	0.43	0.41	0.71	0.84	0.37
Castaño Overo5	ar106	-41.90	-71.48	16 (32)	1539-1991	0.84	0.43	0.34	0.66	0.94	0.42
Castaño Overo6	ar107	-41.90	-71.48	13 (21)	1562-1991	0.93	0.43	0.30	0.67	0.88	0.35
Castaño Overo7	ar108	-41.90	-71.48	11 (21)	1572-1991	0.78	0.43	0.33	0.70	0.92	0.43
**Regional Chrono Bariloche**	**RM Bariloche**			**342 (710)**	**1539-2011**	**0.93**	**0.46**	**0.32**	**0.65**	**0.99**	**0.19**

MW mean ring-width, SD ring-width standard deviation, MS mean sensitivity, AC first-order autocorrelation, EPS expressed population signal, rbar mean correlation among series. 'arXXX' sites (16 sites) are directly available in the International Tree-Ring Databank (ITRDB).

Table S2.- Summary of the dendrochronological statistics of *A. chilensis* chronologies for the selected sites.

Site	Code	Lat (N)	Long (+E / -W)	Trees (radii)	Time span (AD)	MW (mm)	SD (mm)	MS	AC1	EPS	rbar
Melado & Rancho Maule	mau	-35.52	-71.00	88 (154)	1270-2014	0.69	0.39	0.24	0.79	0.96	0.34
Laja	laj	-37.20	-71.32	58 (105)	1418-2005	0.62	0.34	0.25	0.76	0.97	0.44
Polcura	pol	-37.40	-71.25	85 (153)	1144-2004	0.60	0.32	0.28	0.74	0.96	0.38
Nitrao	nit	-37.42	-71.18	56 (115)	1339-2005	0.57	0.31	0.29	0.72	0.96	0.35
**Regional Chrono Diguillin**	**RM Diguillin**			**287 (527)**	**1144-2014**	**0.62**	**0.34**	**0.26**	**0.76**	**0.98**	**0.29**
Ralco-Lepoy	ral	-38.40	-71.19	55 (86)	1585-2005	0.76	0.39	0.23	0.81	0.96	0.35
Collunco alto & Cerro la Hormiga	cohor	-39.56	-71.80	44 (78)	1508-2003	0.81	0.40	0.25	0.73	0.95	0.38
Ñorquinco	nor	-39.7	-71.70	50 (102)	1562-2003	0.63	0.33	0.29	0.72	0.95	0.35
**Regional Chrono Temuco**	**RM Temuco**			**149 (266)**	**1508-2005**	**0.73**	**0.37**	**0.26**	**0.75**	**0.98**	**0.29**
La Fragua	fra	-41.4	-71.59	12 (23)	1690-2002	0.98	0.40	0.20	0.75	0.87	0.36
Los Leones	leo	-41.5	-71.9	54 (73)	1539-2003	0.64	0.38	0.23	0.83	0.96	0.39
El Maitén	mai	-41.59	-71.15	5 (13)	1690-1974	1.63	1.00	0.29	0.77	0.82	0.37
Pampa del Toro	pam	-41.32	-71.29	7 (16)	1741-1991	0.79	0.66	0.20	0.91	0.91	0.44
**Regional Chrono Bariloche**	**RM Bariloche**			**78 (125)**	**1539-2003**	**0.83**	**0.48**	**0.23**	**0.82**	**0.96**	**0.30**

Table S3.- Information of climate stations.

	Region	Tmin period	Tmean period	Coordinates	Elevation (m a.s.l.)
Diguillin	Diguillin	1965-2016	1965-1993	36.87°S 71.64°W	670
Ancoa		1965-2013	1965-1993	35.91°S 71.30°W	421
Temuco	Temuco	1950-2018	1955-2018	38.77°S 72.64°W	92
Liucura		1987-2015	--------	38.65°S 71.09°W	1043
Bariloche Aero	Bariloche	1939-2018	1939-2018	41.15°S 71.16°W	840
El Bolsón Aero		2011-2018	2011-2018	41.94°S 71.53°W	341
